# *In Vitro* and *In Vivo* Evaluation of pH-Sensitive Hydrogels of Carboxymethyl Chitosan for Intestinal Delivery of Theophylline

**DOI:** 10.5402/2012/763127

**Published:** 2012-07-01

**Authors:** Hemant Kumar Singh Yadav, H. G. Shivakumar

**Affiliations:** Department of Pharmaceutics, JSS College of Pharmacy, JSS University, Sri Shivarathreeshwara Nagar, Karnataka, Mysore 570 015, India

## Abstract

Chitosan is a natural polymer which has limited solubility. Chitosan gets solubilized at acidic pH but is insoluble at basic pH. In the present study, carboxymethyl chitosan (CMC) was prepared which shows high swelling in basic pH and thus can delay the drug release and can act as matrix for extended release formulation. CMC was characterized by FTIR and NMR. pH-sensitive hydrogels of theophylline were formulated using CMC and carbopol 934. Hydrogels were evaluated for swelling, drug content *in vitro* drug release studies, and *in vivo* studies on rabbit. The swelling studies have shown little swelling in acidic pH 432% at the end of two hours and 1631% in basic pH at the end of 12 hours. The release profile of the formulation I containing CMC and carbopol in 1 : 1 ratio showed sustained release. *In vivo* studies showed that the release of theophylline from the prepared hydrogel formulation (Test) exhibit better prolonged action when compared to (standard) marketed sustained release formulation. The studies showed that the pH-sensitive hydrogel of CMC can be used for extended release of theophylline in intestine and can be highly useful in treating symptoms of nocturnal asthma.

## 1. Introduction

Chitosan is biodegradable and biocompatible polymer. Since chitosan is insoluble in water, the use of chitosan in basic environment is limited and hence delivery of drugs to the intestine is not possible. A derivative of chitosan, that is CMC, is soluble in water [1, 2]. Amphoteric polyelectrolyte hydrogels possessing both positive and negative charges, and many researchers are using amphoteric polyelectrolyte hydrogels to develop controlled delivery systems such as an insulin pump for diabetics, matrices for molecular recognition or separation, and so forth. A lot of research is going on the stimuli-sensitive polymer hydrogels. Among stimuli-sensitive systems, pH or temperature-responsive hydrogels have been extensively studied in the biomedical field, because these two factors can be easily controlled and are applicable both *in vitro* and *in vivo* conditions [3, 4]. CMC is amphoteric polyelectrolyte and has various applications due to its unique chemical, physical, and biological properties, especially its excellent biocompatibility. It is used to prepare wound dressings, artificial bone, and skin, is used as a bacteriostatic agent and blood anticoagulant also. It has also demonstrated good pH and ion sensitivity in aqueous solutions due to abundant –COOH and –NH_2_ groups [5]. Recent studies have shown that CMC has been used in preparation of nanoparticles for treatment of cancer [6, 7]. The use of CMC has also been explored for delivery of antimicrobial agents [8] and proteins [9]. Extended release matrix tablets have been studied using chitosan and carbopol [10].

Authors of this work have previously investigated CMC hydrogels to deliver methylprednisolone [11]. They found that hydrogels show minimal swelling in acidic pH. Considering this behavior of hydrogels the present study was carried out. Carbopol 934 is a polymer which is sensitive to pH and was used in the present study along with chitosan. To combine the advantage of synthetic and natural polymers and at the same time maintain the property of natural polymers such as biodegradation and bioactivity, amphoteric polyelectrolyte hydrogels with pH sensitivity were synthesized with CMC and carbopol 934 in this work. The swelling behavior of the hydrogel under different pH was studied. The release behavior of theophylline was investigated, when it was loaded into the pH-sensitive hydrogels. Theophylline is a antiasthmatic drug and the dosing of theophylline is complicated because it shows extensive variation in bioavailability among patients. About 75% of people with asthma have symptoms that disrupt both the length and depth of their nighttime sleep at least once a week. The number of inflammatory cells in the airways is highest in the early morning, with a peak at 4 AM. In one study, patients with nocturnal asthma were found to have a 20% decrease in lung function overnight compared with 4% in nonasthmatics. Hencem changing the timing or dosage of the medications may improve symptoms one experiences at night. Many different asthma medications have been specifically studied for their effectiveness at night.

 Theophylline comes in both short-acting and slow-release formulations, once or twice a day. It comes as a pill or in granules which should be swallowed whole, so as not to release too much medication at one time. The main drawback of this type of dosage form is that when blood levels are too high, unpleasant side effects may occur, such as nausea, vomiting, abdominal pain, jitteriness, insomnia, rapid or irregular heartbeat. Theophylline, if delivered to the intestine can be useful in treatment of nocturnal asthma and a single dose of a slow release or extended release theophylline preparation given at night, may provide effective control of nocturnal asthma symptoms.

In the present paper, an attempt was made to formulate a pH-sensitive hydrogel from CMC containing theophylline which has not been attempted so far and evaluate it *in vitro* and *in vivo*. The polymer exhibits pH dependent swelling that is, they swell and release the drug depending on pH range, hence sustained or extended drug delivery is possible in the basic environment of gastrointestinal tract. CMC since soluble in water, undergoes extensive swelling in basic pH compared to acidic pH and drug release is maximum in intestine, thus it can be highly helpful in controlling symptoms of nocturnal asthma.

## 2. Experimental

Theophylline was gift sample from Strides Arco lab Limited, Bangalore. Chitosan (MW = 3.5 × 10^5^, >80% deacetylated) was purchased from Sigma Aldrich, USA. Carbopol 934 was purchased from Loba Chemie Pvt. Ltd., India. All other chemicals were of analytical grade. There is no conflict of interests for any financial gain, as the chemicals were purchased from the companies.

### 2.1. Preparation of CMC

Chitosan solution was prepared in acetic acid and methanol, and acetic anhydride was added under stirring at room temperature. The mixture was stored overnight at room temperature to give a rigid gel. The prepared gel was agitated with 0.5 M NaOH in ethanol at room temperature overnight. The solution was precipitated by addition of concentrated NH_4_OH solution and filtered. The product was washed with 75% ethanol and dried in dessicator. The product which is formed after drying is N-acetyl chitosan. N-acetyl chitosan was suspended in 50% NaOH and kept at −20°C overnight. The product was transferred to 2-propanol and chloroacetic acid was added in portions under stirring. After stirring at room temperature for 2 hr, the reaction mixture was heated to 60°C for another 2 hr. Dialysis was carried out against deionized water for 3 days; the product obtained was dried in dessicator. The dried product obtained was CMC [12–14].

### 2.2. Preparation of Hydrogel

CMC solution was prepared in distilled water under stirring at 5000 rpm for 30 min. Theophylline was added to CMC solution and the solution was stirred for 15 min. Carbopol 934 dissolved in 1.75 M acetic acid was added to CMC solution gradually under stirring. The turbid dispersion obtained was immediately poured into petri dish and kept overnight for cross linking at room temp. The hydrogel obtained was cut into 1 cm × 1 cm pieces and dried for 24 hr under vacuum. The dried hydrogel was crushed and passed through sieve #60/85. The hydrogel particles retained on sieve #85 were used for further studies. The formulation chart of different formulations is given in Table 1 [11].

### 2.3. Fourier Transforms Infrared (FTIR) Spectral Analysis

The prepared hydrogel was subjected to FTIR analysis by KBr pellet method using fourier transform infrared (FTIR) spectrophotometer (Perkin Elmer, spectrum-100, Japan). This was employed to ascertain the compatibility of drugs with the excipient.

### 2.4. Scanning Electron Microscopy (SEM)

SEM studies were carried out on hydrogel samples after coating with gold palladium on a scanning electron microscope, Joel SEM analysis instrument, Japan.

### 2.5. Differential Scanning Calorimetry

Differential scanning calorimetry was performed on a pure sample of theophylline and its formulation, using Shimadzu DSC-50 apparatus. Differential scanning calorimetric thermograms of 2 to 3 mg samples were recorded at a heating rate of 5°C/min in an open aluminium pan over the range of 25°C–300°C.

### 2.6. Nuclear Magnetic Resonance (NMR)

Nuclear magnetic resonance studies were carried out on CMC to determine whether the conversion of chitosan has occurred to CMC using C^13^ NMR and using an NMR spectrometer (DSX-300, Bruker, India). The solid state (without solvent) NMR was done at 75 MHz.

### 2.7. Estimation of Theophylline Content of the Hydrogels

Here an amount of hydrogels containing 20 mg of theophylline was placed in 7.4 pH phosphate buffer solution, for 24 hours. In the 7.4 pH phosphate buffer solution the hydrogels swell and the drug is released. At the end of 24 hours, amount of theophylline present in 7.4 pH phosphate buffer is determined spectrophotometrically at 272 nm. The method was validated for linearity, accuracy, and precision. The method obeyed Beer's law in the concentration range 2–14 *μ*g/mL.

### 2.8. Swelling Studies

The pH-dependent swelling property of hydrogel was studied by immersing the dry hydrogels in aqueous solutions of the pH 1.2 HCl buffer for 2 hr and then in pH 7.4 phosphate buffer for another 8 hr. After regular intervals of time, hydrogels were removed from the aqueous solution, excess surface water was removed with filter paper, weighed, and returned to the same container until equilibrium was observed [13, 15]. The degree of swelling (*W*_*t*_) was calculated at different times by means of following equation:

(1) S=(weight  of  swollen  hydrogel−weight  of  dry  hydrogel)×100weight  of  swollen  hydrogel.

### 2.9. *In Vitro* Drug Release Studies

*In vitro* drug release from the hydrogels was carried out in triplicate at 37 ± 0.1°C in USP XXII dissolution apparatus type II (six basket dissolution tester-USP XXII,TDT-08L, Electrolab, Mumbai, India) at a rotation speed of 50 rpm. A sample of hydrogel equivalent to 300 mg of theophylline was used in each test. Drug release from the hydrogel was studied in 900 mL of dissolution medium (2 hr in pH 1.2 HCl buffer and 10 hr in pH 7.4 phosphate buffer). Sample of dissolution fluid was withdrawn through a filter (0.45) *μ*m at every hour and was assayed at 272 nm for theophylline content using a Shimadzu UV-1700 double beam spectrophotometer [13]. The release data obtained were fitted into korsmeyer-peppas equation, log⁡% *R* = log⁡*K* + *n*log⁡*t*, where *R* is the amount of drug released in given time *t*, *K* is the release rate constant, and *n* is the time exponent. A graph of log⁡% *R* v/s log *t* was plotted. The intercept on *Y*-axis gave the value of *K*, the release rate constant and the slope the value of *n*, the time exponent. To determine the release mechanism, the parameter *n* and *k* were used.

### 2.10. *In Vitro* Wash-Off Test for Mucoadhesion

About 50 hydrogel particles were taken for the study and spread over the sheep's intestinal mucosa (2 × 2 cm) taken as a biological substrate for studying mucoadhesive nature of the hydrogels. The prepared hydrogel was passed through sieve number 20; the particles which were retained on the sieve were coarser and were counted and taken for the study. The instrument designed was, disintegration apparatus USP, the 6 tubes were removed and the mocosa was fixed to the base of the apparatus. The medium chosen was 7.4 pH phosphate buffer, every 5 min interval hydrogel particles adhering to the mucosa were counted. The study was carried out for 30 min [16].

### 2.11. *In Vivo* Studies

The *in vivo* release studies were conducted on albino rabbits weighing 2.5–3 kg. The animals were divided into two groups of 6 rabbits each as a standard and the other as test. A written approval was obtained from the Institutional ethical committee of JSS Medical College and Hospital and JSS College of Pharmacy, Mysore, India. Detailed verbal and written information on the study was provided to the Veterinary Surgeon, Central Animal Facility, JSS Medical College and Hospital and permission was obtained. The animals were fasted for 12 hours before the capsules were introduced into the oesophagus and washed using 5 mL of distilled water in order to avoid the possible damage caused by chewing. Blood samples were collected from ear vein at 1, 2, 4, 8, 16, 24 hr after the oral administration [17].

The blood samples were centrifuged and plasma was stored at −20°C for further analytical determination. To the above samples, isopropyl alcohol was added and vortexed for 30 sec. The drug was extracted with 2 mL of chloroform and vortexed at high speed for 1 min. After centrifugation at 1000 rpm for 5 min, the organic layer was evaporated and the residue was reconstituted with 100 mL of the mobile phase. This solution was injected into the HPLC system for analysis. The instrument used was Shimadzu LC-2010AHT. Acetonitrile (7.5%) in 0.2% acetic acid solution was used as the mobile phase. Column used was C_18_. The temperature was kept ambient. Injection volume was 20 *μ*L at 1.5 mL/min flow rate [18]. The sample run time was 8 min.

 The *in vivo* studies were conducted on prepared optimized hydrogel formulation (Test) and on marketed sustained release formulation Theobid SR tablet from Cipla (Standard).

### 2.12. Stability Studies

Stability studies were conducted on optimized formulation of CMC hydrogels to assess their stability with respect to their physical appearance, drug content, swelling, and drug release characteristics after storing them at 25°C/(RH) 60%, and 30°C/(RH) 65% as per ICH Q_1_A (R_2_) regulations for 6 months.

## 3. Results and Discussion

### 3.1. Preparation of CMC

Chitosan is a unique polysaccharide derived from deacetylation of chitin. When chitosan is changed into O-carboxymethyl chitosan (O–CMC) by introducing –CH_2_COOH groups onto –OH along chitosan molecular chain, an amphoteric polyelectrolyte containing both cationic and anionic fixed charges was prepared [16]. By varying degree of deacetylation and carboxymethyl group substitution of the chitosan, we can obtain CMC. Carboxymethyl substituents were observed on amino and hydroxyl sites on the surface of modified chitosan. The preparation was carried out in two steps. First, N-acetyl chitosan was prepared using acetic anhydride, then carboxymethylation was done to get CMC. As given in literature at 50% concentration NaOH gives better degree of substitution, hence, 50% concentration was used [16]. The prepared CMC is white-colored free-flowing powder and shows good solubility in both water and organic solvents, which extends its range of applications. CMC shows characteristic behavior with pH. This property and water solubility was used in preparing pH-sensitive hydrogels in the present work.

### 3.2. Preparation of CMC Hydrogels

CMC is amphoteric in nature and contains positively charged groups, they interact with negatively charged carboxylic groups of Carbopol and form interpolyelectrolyte complexes (IPECs) which were stabilized by cooperative ionic bonds. Moreover, interpolymer interactions were possible between countercharged groups in the own macromolecule and of course between copolymer chains of different macromolecules. Due to its good solubility in wide range of pH values, the CMC solution could be readily blended with polyacrylic acid solution and homogenous hydrogels were obtained. The cross-linking was carried out at room temperature. Different formulations were prepared by varying the concentration of CMC by keeping the concentration of polyacrylic acid constant from F1–F5. For the formulation F1 to F5 the concentration of carboxymethylchitosan was increased gradually from 1%, 1.25%, 1.5%, 1.75%, and 2%, respectively.

### 3.3. FTIR Studies

**Figure 1:**
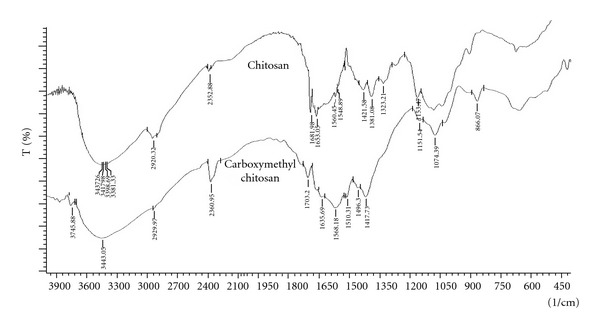
FTIR spectra of chitosan and CMC.

**Figure 2:**
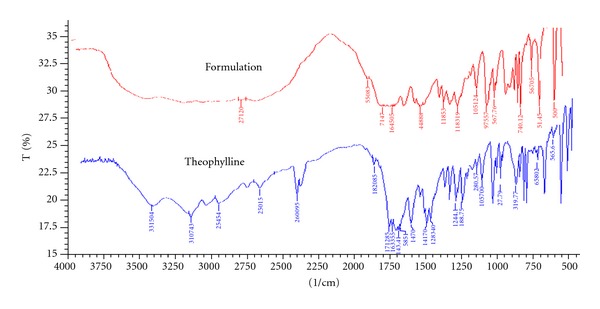
FTIR spectra of theophylline and hydrogel.

FTIR studies were carried out for carboxymethylchitosan and chitosan, and the spectra are given in Figure 1. Spectra showed signals of nonmodified chitosan at 1,653 and 1,560 cm^−1^ for the C–O stretching (amide) and N–H bending (amine), respectively. Other characteristic peaks of chitosan O–H stretch, C–H stretch, and C–O stretch were present at 3,400–3,600, 2,800–2,900 and 1,020–1,180 cm^−1^, respectively. The spectra of carboxymethylchitosan are similar to that of the original chitosan with a new peak appearing at 1,703 cm^−1^, which is assigned to the carbonyl groups. This confirmed the conversion of chitosan to carboxymethylchitosan. Carboxymethylchitosan showed the disappearance of the  –NH_2_ associated band at 1595 cm^−1^, which can be ascribed to characteristic vibration deformation of the primary amine N–H and the appearance of some new intensive peaks at 2922–2852, 1466, and 721 cm^−1^ which can be attributed to the methyl groups and the long carbon segment of the quaternary ammonium salt. Characteristic peaks of the hydroxyl and second hydroxyl groups between 1152 and 1030 cm^−1^ did not change. Theophylline (pure drug) and hydrogel formulation were subjected for FTIR spectroscopic to ascertain whether there is any interaction between the drugs and polymers used. The characteristic peaks of the pure drug were compared with the peaks obtained for their formulation. It was observed that similar characteristic peaks appear with minor differences, at 1654 cm^−1^ (C=O stretching amide), 1596 cm^−1^ (C=C stretching aromatic), 1307 cm^−1^ (C–O stretching) for theophylline and for the formulation as shown in Figure 2. Hence, it can be concluded that the drug is in free state and there is no interaction between drug and polymer used.

### 3.4. C^13^ NMR Studies

**Figure 3:**
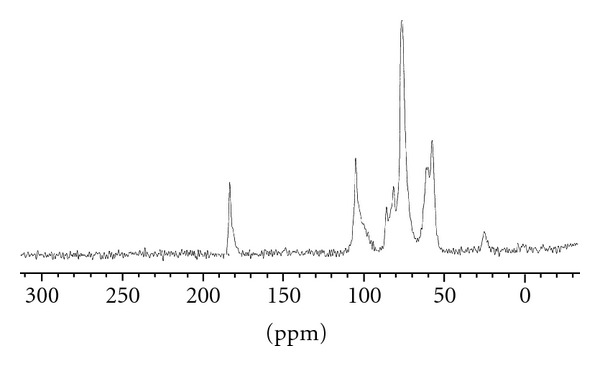
NMR spectra of CMC.

The C^13^ NMR of chitosan was studied as given in literature and compared with the spectra of CMC. Chitosan spectra show peaks at 177.9 ppm and at 25 ppm, which are assigned to the carbonyl carbon of –COCH3 and the methyl carbon (–CH3), respectively. These signals are less intense than the other signals. The signal at 101.3 ppm is assigned to the hydrogen bonded to carbon of chitosan and the signals in 59.6 ppm, 73.1 ppm, 81.1 ppm, 78.6 ppm, and 64 ppm are assigned to carbons of glucopyranose [19]. The C^13^ NMR of carboxymethylchitosan was carried out and shown in Figure 3. Spectra show the signal shifted from 101.3 ppm to 105.9 ppm because of the electron-withdrawing effect of the carboxymethyl substituents. Since various different units occur in the structure of carboxymethylchitosan, many of the signals in the spectrum of chitosan appear split in the spectrum of carboxymethylchitosan. Thus, the signals at 60.1 ppm, 73.8 ppm, 73.2 ppm, 82.2 ppm 78.2 ppm, and 63.9 ppm are split and shifted in relation to those detected in the spectrum of the parent chitosan. The signal observed at 180.7 ppm is assigned to the carbonyl carbons of carboxymethyl groups while the one detected at 177.9 ppm corresponds to the carbonyl carbon of –COCH3 of the parent chitosan. The methylene groups (–CH2), carbons give rise to the signals at 53 and 57.4 ppm, respectively. However, no signal was detected at 53 ppm in the spectrum of carboxymethylchitosan Figure 3 and the weak signal at 58.4 ppm can be probably assigned to the methylene (–CH2) bonded to the amino group (–NH) [19]. These features are taken as evidence that the carboxymethylation occurred at the hydroxyl as well as in the amino groups of chitosan which is also supported by FTIR studies. The spectra are shown in Figure 3.

### 3.5. Scanning Electron Microscopy

**Figure 4:**
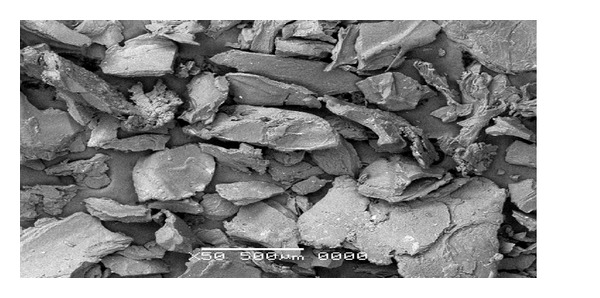
SEM photograph of hydrogel particles.

Scanning electron microscopy was carried out in order to study surface morphology, texture, and porosity of hydrogels. The SEM photograph of hydrogel clearly showed the rugged nature of hydrogel particles. The SEM photograph is shown in Figure 4.

### 3.6. Differential Scanning Calorimetry

DSC studies of pure drug and F1 were studied to determine the possible interaction between the drug and the hydrogel. Thermogram of theophylline has shown a sharp endothermic peak at 272.41°C, which corresponds to its melting point. F1 formulation hydrogel also showed endothermic peaks at 271.59°C. The evaluation of thermograms obtained from DSC revealed no interaction between the drug and polymers used, as there was no significant change in the melting point of theophylline. The obtained results are shown in Table 2.

### 3.7. Drug Content

The test for drug content was carried out to ascertain that the amount of drug in the formulation. From the results obtained, it can be inferred that there is proper distribution of theophylline in the hydrogels. The drug content analysis showed that the drug is uniformly distributed in the range of 74.5–88.6% of the total amount of the drug added in different formulations.

### 3.8. Swelling Studies

**Figure 5:**
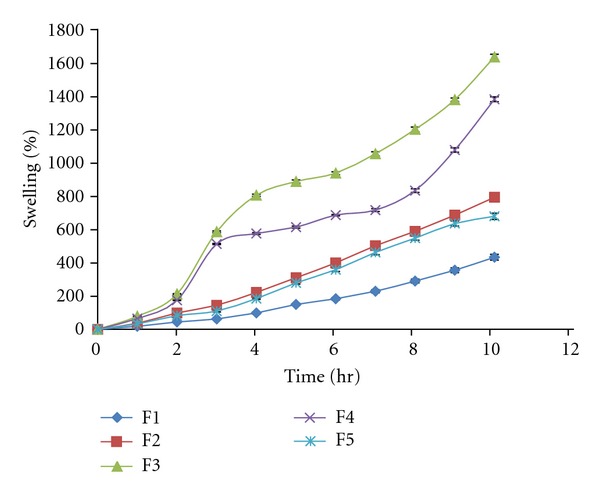
% swelling of F1–F5 hydrogels for 10 hr.

The swelling behavior of CMC was studied. Swelling studies were conducted for 12 hrs but swelling did not show significant change after 10 hrs until 12 hrs hence data for 10 hrs is presented. The swelling in water mainly depends on the osmotic pressure difference between inside the gel and the surroundings caused by redistribution of mobile ions. The swelling was observed to be more at basic pH due to increase in the number of mobile ions inside the gel and large osmotic pressure leads to swelling. The results for swelling studies are shown in Figure 5. % swelling was found in range of 432.75–1631.56. The results indicate that with an increase in pH from 1.2 to 7.4, a considerable increase in swelling was observed for all the hydrogel formulations, which may be due to the dissociation of the –COOH groups of CMC, thereby increasing the osmotic pressure inside the hydrogels resulting in increased swelling [16]. Swelling increased when ratio of CMC was changed till 1.5 : 1 with polyacrylic acid, that is, formulation F1–F3 but further increase in ratio, that is, 1.75 : 1 and 2.0 : 1 (F4 and F5) swelling decreased. This shows that CMC and polyacrylic acid have synergistic effect till certain ratio as a result of which swelling increases but further increase in CMC amount resulted in reduced water uptake which may be attributed to the antagonistic effect resulting in decrease swelling. Swelling strongly depends on the extent of cross-linking. At lower cross-linking, the network is loose with a greater hydrodynamic free volume, so that the chains can accommodate more of the solvent molecules resulting in higher swelling. In this study it was found that the swelling was increased when the pH was changed from acidic to basic, and it conforms that the prepared hydrogel was sensitive to pH. The effect on swelling with time is shown is Figure 5.

### 3.9. *In Vitro* Drug Release Studies

**Figure 6:**
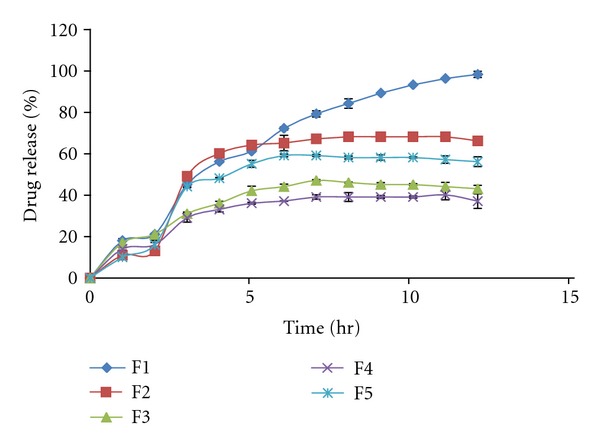
*In vitro *drug release of different formulations.

 The *In vitro* release studies were carried out for all the formulations in both acidic and basic media. The release studies were carried in the pH 1.2 HCl buffer for the first two hours, to mimic the acidic conditions prevailing in the stomach. For the next 10 hours, the release studies were carried out in pH 7.4 pH phosphate buffer, to mimic the alkaline conditions of the intestine. For the initial 2 hours that is, in the pH 1.2 HCl buffer, the percentage drug release was found to be low in all the cases; this can be attributed to the fact that the hydrogel swells less in the acidic medium. When the dissolution medium was changed to pH 7.4 pH phosphate buffer, the release was found to increase, with time. The drug release showed effect on basis of swelling. Drug release decreased, from F1–F3 and again increased for F4-F5. The effect observed is based on swelling, as swelling increases the drug release decreases and again when swelling decreases drug release increases as shown in Figure 6. On the basis of above studies conducted F1 was chosen as optimized formulation as it showed desired sustained-release profile along with other drug content and swelling studies results. All formulations present an initial burst effect. It may be attributed to diffusion of the drug caused by rapid gel swelling and also the release of drug adsorbed towards the surface of the gel matrix. The % drug release was found in range of 37.21–98.47 at the end of 12 hrs. The data obtained from *in vitro* release studies was fitted into various mathematical models. The regression coefficient, *R* ^2^ obtained is given in Table 3. *R* = regression coefficient. The value of *n* determined from korsmeyer peppas equation was in the range of 0.5–0.7, which indicates the drug release from the hydrogels followed non-fickian or anomalous mechanism (relaxation controlled).

### 3.10. *In Vitro* Wash-Off Test for Mucoadhesion

The mucoadhesive study showed that all the hydrogel particles get detached from the mucosa within 20 min. This study shows that carboxymethylchitosan does not have a good mucoadhesion property when compared to well-known mucoadhesive strength of parent chitosan. This can be attributed to the better solubility of CMC in water and organic solvents.

### 3.11. *In Vivo* Studies

**Figure 7:**
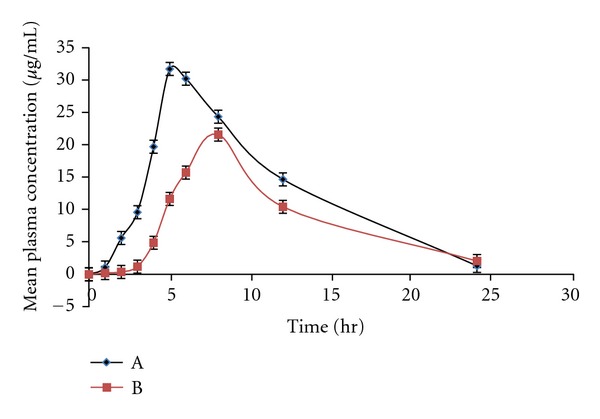
Mean plasma concentrations time profiles of theophylline product A and B.

*In vivo* studies were carried out for Theobid SR tablet from Cipla (product A) and theophylline loaded hydrogels of CMC both containing 300 mg of theophylline on albino rabbits. Blood samples were withdrawn at different time intervals and plasma concentrations of theophylline were estimated, the profile is presented in Figure 7. Statistical comparison of the mean values of pharmacokinetic parameters derived for both products A and B are given in Table 4. From the data obtained, it may be observed that after oral administration, peak plasma concentration *C*_max⁡_ of 12.34 ± 2.42 *μ*g/mL was observed for product A and 09.69 ± 4.12 *μ*g/mL for product B. From the comparison of mean values of plasma concentrations of product A and B, it was observed that product B has lower plasma concentrations. It was observed that the plasma concentration of theophylline in all animals after 24 hrs of oral administration was below 20 *μ*g/mL for both products. It was also observed from the studies that the therapeutic concentration range of theophylline maintained for about 24 hrs following a single oral dose administration for both products.

From the data obtained, it may be observed that the time taken to reach peak plasma concentration *T*_max⁡_ was 5.0 ± 0.81 hrs for product A and 6.0 ± 0.75 hrs for product B. Mean elimination rate constant Kel was found to be 0.08410 h^−1^ for product A and 0.07813 h^−1^ for product B. Similarly mean elimination half life *t*_1/2_ for product A was 8.24 ± 4.7 hrs and for product B 8.87 ± 5.74 hrs. The mean AUC_0-24_ values for product A was 101.73 ± 16.5 *μ*g·hr/mL and for product B 123.17 ± 21.5 *μ*g·hr/mL. The lower *C*_max⁡_, prolonged *T*_max⁡_, of theophylline in rabbits indicated that the drug release from the product B is slow, thereby providing a prolonged and controlled *in vivo* delivery of the drug. These *in vivo* absorption characteristics are in confirmation with the observed *in vitro* drug release rate of the drug from the hydrogel.

### 3.12. Stability Studies

**Figure 8:**
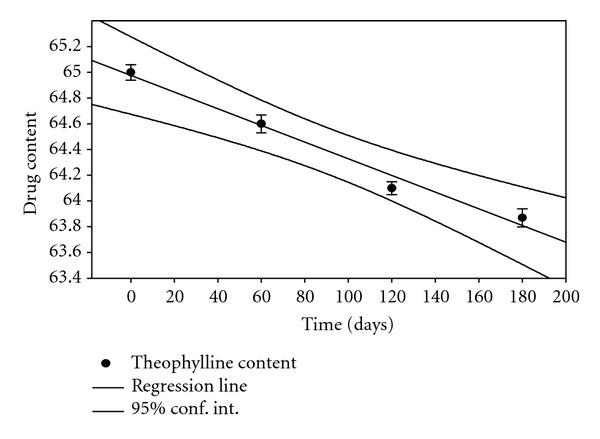
Theophylline content at 25°C/(RH) 60%.

**Figure 9:**
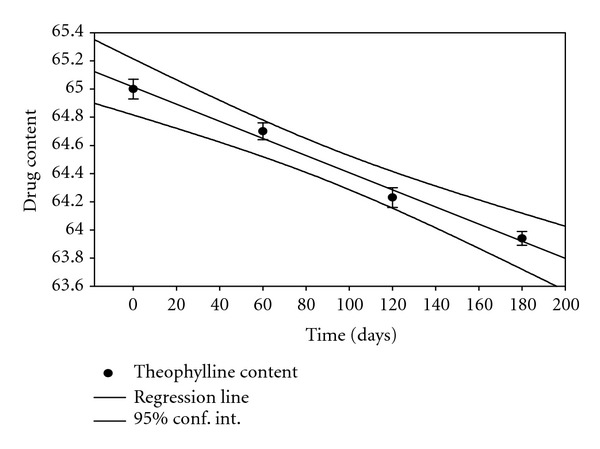
Theophylline content at 30°C/(RH) 65%.

Stability studies were conducted for different formulations for a period of 6 months. At specific time intervals, the samples were tested for drug content, swelling, and % drug release. The drug content in samples was tested at 2 different conditions along with 95% confidence limits, using sigma plot software 10.0 as shown in Figures 8 and 9. Stability studies results obtained at various intervals showed that the hydrogel prepared from CMC did not show significant difference in physical appearance at the end of 6 months except in change of colour from light brown to brown. % drug release, % swelling, and mucoadhesion did not change significantly at the end of stability studies. From the data, it can be seen that hydrogels prepared are stable at given two conditions.

## 4. Conclusion

From the results obtained, it may be concluded that the prepared hydrogels were pH-sensitive and the degree of swelling of hydrogel depends on the concentration of cross-linking agent as well as on the pH of the environment. As the theophylline is released highly in basic pH the peak theophylline concentration will be achieved at early morning which will be highly beneficial to the patients suffering from nocturnal asthma. The *in vitro* and *in vivo* studies showed that the drug release is slowed down and prolonged which is better than commercial available formulation and hence better. Thus, the hydrogel prepared using CMC can be used to deliver theophylline in sustained manner and can be used effectively against nocturnal asthma; CMC can also be useful for the delivery of drugs which are unstable in acidic pH.

## Figures and Tables

**Table 1:** Different formulation prepared of theophylline hydrogels.

Sl. No	Ingredient	F1	F2	F3	F4	F5
	↓ Ratio →	1 : 1	1.25 : 1	1.5 : 1	1.75 : 1	2 : 1
1	Theophylline (mg)	300	300	300	300	300
2	Carboxymethylchitosan (mg)	1000	1250	1500	1750	2000
3	Carbopol 934 (mg)	1000	1000	1000	1000	1000
4	Distilled water (mL)	25	25	25	25	25
5	1.75 M Acetic acid (mL)	25	25	25	25	25

**Table 2:** DSC data of pure drug and F1 formulation.

Sl. No.	Drug and with hydrogel	*T*_0_ (^°^C)	*T*_*m*_ (^°^C)	*T*_*c*_ (^°^C)
**1**	Drug	264.82	272.41	280.19
**2**	F1 formulation	267.41	272.09	279.66

**Table 3:** Model fitting for different formulations.

	*R* ^2^				
	F1	F2	F3	F4	F5
Zero order	0.763	0.682	0.714	0.701	0.670
First order	0.861	0.737	0.752	0.737	0.00
Higuchi	0.902	0.829	0.901	0.890	0.832
Peppas	0.957	0.910	0.950	0.963	0.897
Best fit model	Peppas	Peppas	Peppas	Peppas	Peppas
Diffusion exponent Peppas model	*n* = 0.6937	*n* = 0.5826	*n* = 0.784	*n* = 0.752	*n* = 0.652

**Table 4:** A statistical comparison of the mean values of pharmacokinetic parameters of product A and B.

Parameters	Product A	Product B	*P*
*C*_max⁡_	12.34 ± 2.42 *μ*g/mL	09.69 ± 4.12 *μ*g/mL	<0.05
*T*_max⁡_	5.0 ± 0.81 hr	6.0 ± 0.75 hr	<0.05
Kel	0.08410 h^−1^	0.07813 h^−1^	<0.05
*t*_ 1/2_	8.24 ± 4.7 hrs	8.87 ± 5.74 hrs	<0.05
AUC_0–24_	101.73 ± 16.5 *μ*g·hr/mL	123.17 ± 21.5 *μ*g·hr/mL	<0.05

## References

[1] Kumar R, Majeti NV (2000). A review of chitin and chitosan applications. *Reactive and Functional Polymers*.

[2] Amiji M, Hejazi R (2003). Chitosan-based gastrointestinal delivery systems. *Journal of Controlled Release*.

[3] Hoffman AS (2002). Hydrogels for biomedical applications. *Advanced Drug Delivery Reviews*.

[4] Guo B, Yuan J, Yao L, Gao Q (2007). Preparation and release profiles of pH/temperature-responsive carboxymethyl chitosan/P(2-(dimethylamino) ethyl methacrylate) semi-IPN amphoteric hydrogel. *Colloid and Polymer Science*.

[5] Chen XG, Wang Z, Liu WS, Park HJ (2002). The effect of carboxymethyl-chitosan on proliferation and collagen secretion of normal and keloid skin fibroblasts. *Biomaterials*.

[6] Mathew ME, Mohan JC, Manzoor K, Nair SV, Tamura H, Jayakumar R (2010). Folate conjugated carboxymethyl chitosan-manganese doped zinc sulphide nanoparticles for targeted drug delivery and imaging of cancer cells. *Carbohydrate Polymers*.

[7] Dev A, Mohan JC, Sreeja V (2010). Novel carboxymethyl chitin nanoparticles for cancer drug delivery applications. *Carbohydrate Polymers*.

[8] Wang X, Liu B, Ren J (2010). Preparation and characterization of new quaternized carboxymethyl chitosan/rectorite nanocomposite. *Composites Science and Technology*.

[9] El-Sherbiny IM (2010). Enhanced pH-responsive carrier system based on alginate and chemically modified carboxymethyl chitosan for oral delivery of protein drugs: preparation and in-vitro assessment. *Carbohydrate Polymers*.

[10] Park SH, Chun MK, Choi HK (2008). Preparation of an extended-release matrix tablet using chitosan/Carbopol interpolymer complex. *International Journal of Pharmaceutics*.

[11] Joshi GB, Ksy H, Singh MN, Shivakumar HG (2011). Development of pH sensitive hydrogel for intestinal delivery of methyl prednisolone using novel chitosan derivative. *International Journal of Pharmacy and Pharmaceutical Sciences*.

[12] Chen XG, Park HJ (2003). Chemical characteristics of O-carboxymethyl chitosans related to the preparation conditions. *Carbohydrate Polymers*.

[13] Lin YH, Liang HF, Chung CK, Chen MC, Sung HW (2005). Physically crosslinked alginate/N,O-carboxymethyl chitosan hydrogels with calcium for oral delivery of protein drugs. *Biomaterials*.

[14] Dong AJ, Feng MH (2008). Synthesis and properties of O-carboxymethyl chitosan/methoxypoly(ethylene glycol) graft copolymers. *Journal of Materials Science*.

[15] Chen L, Tian Z, Du Y (2004). Synthesis and pH sensitivity of carboxymethyl chitosan-based polyampholyte hydrogels for protein carrier matrices. *Biomaterials*.

[16] Chowdary KPR, Srinivasa Rao Y (2003). Preparation and evaluation of mucoadhesive microcapsules of indomethacin. *Indian Journal of Pharmaceutical Sciences*.

[17] Muskó Z, Pintye-Hódi K, Gáspár R (2001). Study of in vitro and in vivo dissolution of theophylline from film-coated pellets. *European Journal of Pharmaceutics and Biopharmaceutics*.

[18] Om P, Rajat R, Sethi PD (2008). *Quantitative Analysis of Drugs in Pharmaceutical Formulation*.

[19] Abreu S, Campana F, Fernanda R (2005). Preparation and characterization of carboxymethyl chitosan. Polímeros. *Ciência e Tecnologia*.

